# The Healer's Art: Remembering Our Professional Lineage in Community through the Cultivation of Individual Core Values

**DOI:** 10.15694/mep.2018.0000194.1

**Published:** 2018-09-04

**Authors:** Sarah Stender, Sarah Stender

**Affiliations:** 1 UCSF Fresno; 2 LSU Health Science Center

**Keywords:** medical education, professionalism, humanities, hidden curriculum, learning climate, burnout, electronic medical record, artificial intelligence

## Abstract

This article was migrated. The article was marked as recommended.

**Introduction**: There is both patient and provider dissatisfaction with the climate of healthcare delivery. Upon review, this is found to be at least in part attributable to the mechanization of health care, which often involves more computer interaction than hands-on care. Despite rising costs, the physical exam is replaced by lab tests and radiologic studies, generating more cost. The time-honored respect for a carefully obtained history from the patient is replaced by a computer check-box template. The humanity of both physician and patient are marginalized, with increased potential for both diagnostic and therapeutic compromise. Though access to medical information about disease is possible with bioinformatics, artificial intelligence cannot substitute for analysis by an informed, attentive, and properly educated physician. The process of healing must begin with the first patient visit - and the presence of an informed, compassionate, and fully attentive physician.

**Objective**: To describe the history of medical educators’ grappling with this problem through 3 landmark articles over a 100-year period. To illustrate the challenges of the climate of medical education. To offer some educational strategies (with examples of successful programs) to teach physicians using the Humanities. To illustrate that the art and science of medicine are synergistic, not dichotomous.

**Methods**: Two educational theories ripe for use: Chickering and the Discovery Model, and Osler’s recommended bedside reading list, exemplary programs that are being used currently (and over the last 25-plus years) to emphasize the importance of both the science and the practice of medicine in an effort to optimize the medical climate.

**Conclusion**: The problem of physician burnout and patient dissatisfaction is being addressed in the medical literature, by regulatory societies devoted to physician wellness and by medical educators. This is nevertheless a challenge given the current electronic climate (with bioinformatics and artificial intelligence) and revenue-focused agendas of practice management business people.

**Results**: With an awareness of the need for emphasis on the humanities coupled with an historical perspective over the last 100 years, a spirit of hope can be provided to both physician and patient from the lineage of the medical profession, which also is a legacy for our medical students.

## “Wherever the art of Medicine is loved, there is also a love of Humanity.” Hippocrates, the father of modern Medicine, 460-375 BC

There is, and has been for years, a tension between the science and the art of medicine. This tension exists despite the fact that the properly educated physician is expected to have grounding in a college education with focus on the liberal arts along with the scientific requirements for admission.

Medical school admissions committees must carefully consider which candidates have the necessary aptitude, as well as basic grounding in scientific inquiry coupled with broader study of the arts -- usually within a school of “Arts and Science.” Depending upon the undergraduate school, these liberal arts courses may be distributed in Arts and Humanities, Social Sciences, Science and Engineering and Applied Sciences. With the rise of computers, and the immediate availability of knowledge (not necessarily understanding) -- coupled with an exponential explosion of scientific knowledge -- the methods of educating have become increasingly mechanized and artificial, bordering on an application of “artificial intelligence.”

How then does our “system” cultivate morality in the context of ethical dilemma, compassion despite demands for rapid delivery of care, and intellectual curiosity in the face of “canned” answers from internet sites that give lists of differential diagnoses without benefit of nuanced aspects of the patient’s particular condition and situation? Do these systems of modern care cultivate intellectual curiosity and compassionate, individualized treatment plans, or do they foster mechanistic processing of patient illness scripts that might just as easily have been provided by a computer program? Indeed, there are now web services from which patients can order their own lab tests and prescriptions without ever seeing a physician.

The science of medicine is imprecise, and compassionate care in healing, with its elusive nature, is an art. Though there are many more treatments now available (e.g. monoclonoal antibodies, CRISPR techniques, regenerative stem cell therapies) as the pathogenesis of disease is further elucidated, there continue to be many disease states for which we have neither pathophysiologic understanding nor medicines -- much less cures. Many treatments are problematic, with a risk of further worsening of disease and untoward side effect - a situation exacerbated by any lack of long term longitudinal follow up. There are moral and ethical issues surrounding the giving of the treatments themselves, including questions involving financing and dispensing drugs in the face of dwindling resources.

Because of the need to translate basic scientific inquiry into patient care, the science and practice of medicine remains complex despite our many advances.

Much delivery of care focuses the physician’s attention upon a computer. The physician is not fully present to the patient. Who then is the patient? Is it the computer or is it the human being presenting to us -- or to disinterested assistants without investment who may or may not have the powers of observation, the analytical problem-solving ability, the extensive scientific training, and the exposure to the humanities that has been traditionally required and expected of the physician. What of the diagnoses that cannot be reached by a search in “Dr. Google” nor found in an illness script? Do we listen to the narrative of the patient’s words (the history) and his body (the physical exam) or do we click templated boxes and order labs and radiologic studies based on algorithms that may or not lead to the correct solutions? Is the patient a problem to be solved/processed/billed or a human being to be healed/nurtured/cherished/honored?

Is it a surprise then, that the joy and privilege of practicing medicine so delivered is undermined by patient dissatisfaction, resulting in nonadherence and disengagement from care, coupled with physician burnout -- including attrition from the field, depression, substance use, and even suicide?

There is a reason that courses such as the “Healer’s Art Program,” including the writing of a personal Hippocratic oath, (1990), and the Arnold P. Gold foundation White Coat Ceremony -- with the recitation of the Hippocratic Oath, and emphasizing the sacred nature of investiture in the white coat -- have taken root. There is a reason that there are opportunities as well as requirements for service projects, and that there is a rise in the offering of arts and humanities courses, wellness curricula, campus wide book readings focusing upon moral and ethical dilemmas in the context of medicine. International societies (such as the Society for Adolescent Health and Medicine) have for years had the Spirituality Special Interest Group. Medical journals have focused on integration of the arts (e.g. the Journal of the American Medical Association with classical art work on the cover and the regular column “A Piece of My Mind,” in Pediatrics, “Ethics Rounds,” in Annals of Internal Medicine, “On Being a Doctor,” and in the New England Journal of Medicine, “Perspectives,” to name just a few - interspersed with poetry, essay, reflection, and revelation of the shared suffering of physician and patient.)

## “Past things are planted in you that they may out of you like gardens rise.” --Ranier Maria Rilke

Education (from the Latin: *e, ex,*out of, *ducere,*to lead) of the physician begins early in life, manifest as moral, ethical, and spiritual development. Thus, it is no surprise that programs such as the Healer’s Art (Remen Institute for the Study of Health and Illness-RISHI) rely heavily upon experiential learning: generous listening, reflection, journaling, poetry, music, mindfulness, and drawing in an effort to awaken the instinctual humanity that calls us into the science and practice of medicine. Such learning is reminiscent of childhood in which crayons and paper, the use of pen and paper rather than computers, are the norm. Further discussion about this will follow below.

Though Hippocrates, stated that the art of medicine goes hand in hand with love of humanity, that a suffering patient inspires science, we continue to grapple with the balance.

This tension between the science and art of medicine is well illustrated in three publications: The Flexner Report (1910), “Requiem or Reveille: The Clinician’s Choice” (1963, about fifty years later), and “American Medical Education 100 Years after the Flexner Report ” NEJM, 2006, Cooke, Irby, *et al.*

Early 1900’s: Abraham Flexner and William Osler, MD

Abraham Flexner was not a physician, though his brother Simon was. Simon’s post-graduate work at Hopkins had a focus on microbiology (Shigella flexneri) and perhaps is the reason that Flexner knew William Osler, the father of the medical residency. Osler promoted two parallel areas of bedside training: bedside (patient’s) rounds and bedside (physician’s) reading. The similarity in thought of these two people and their approach to education and practice is apparent in Flexner’s description: “medical curricula sadly deficient in cultural and philosophical background” - a balance to his cry for “science.”

It was Abraham Flexner who became concerned about disparities in medical education; his manifesto, The Flexner Report, had the goal of standardization. Neither Abraham Flexner nor William Osler urged that science was to be promoted at the expense of the humanities. Nonetheless, the seductiveness of “pure science,” (if such truly exists) has caused and perpetuated an imbalance between the two perspectives in their rivalry. During the years following his publication, Flexner lamented this very fact, urging that both perspectives and aspects of care were important. William Osler’s own life and career had been influenced by Sir Thomas Browne’s Religio Medici (also referred to by Kalanathi in *When Breath Becomes Air*). Indeed, Osler, believing that the interspersion of scientific studies with humanities was of utmost importance, recommended the following basics for the properly educated physician: The Bible, Plutarch, Marcus Aurelius, Epictetus, Shakespeare, Montaigne, Miguel de Cervantes, Ralph Waldo Emerson, and Oliver Wendell Holmes.

Osler’s Bedside reading list well illustrates Swanwick’s “Roles for Literature in Medical Education:

An *education* (as opposed to *mere training)*

Ethics and Communication Skills

Development of Personal Value

A Sense of Wonder at Embodied Human Nature

1962: John Romano, MD

About 50 years after the publication of The Flexner Report, the article, “Requiem or Reveille: The Clinician’s Choice,” written by John Romano, MD, the chief of Psychiatry at the University of Rochester, originally delivered as an address at the 73rd annual meeting of the Association of American Colleges, 1962, was published *American Journal of Medical Education*, Vol. 38. July. 1963. The article made an appeal for the humanities in the face of increasing scientific knowledge, which had resulted in an expansion of the material to be mastered, and increased the dichotomy between the humanities and scientific “facts.” Dr. Romano speaks of the rising note of concern among medical leaders and educators over the rift between the medical scientist and the medical practitioner, between the investigator and the healer, between the science and art of medicine.

The University of Rochester was a fertile place of awareness where Dr. George Engel worked. He is credited with the birth of the biopsychosocial model of health care, medicine previously having been framed in a reductionist biological model. (We have come a long way now, with focus on ACES and social determinants of biological health -- including the associated development of serious chronic disease.)

2006: M. Cooke, MD, D.Irby, PhD, W. Sullivan, PhD, K Ludmerer, MD

The NEJM article, “American Medical Education 100 Years after the Flexner Report” focuses upon this same plight. It characterizes three rubrics for those providing medical education and service to the field: Clinician-Teacher, Laboratory Scientist, and Physician-Scientist. This article, as it outlines the need for reform in medical education of the 21st century, circles back, echoing Osler’s bedside rounds in this statement: “Cognitive Psychology has demonstrated that facts and concepts are best recalled and put into service when they are taught, practiced, and assessed in the context in which they will be used.” In Osler’s early 20th century model of bedside rounds these words resonate: “He who studies medicine without books sails an uncharted sea, but he who studies medicine without patients does not go to sea at all.”

The climate and context of the medical world has becme less than hospitable (pun intended) and problematic to both provider and patient. Whereas the formal curriculum is composed of lectures and classes, the hidden curriculum is an unspoken, sometimes subconscious present. It is both informal, manifesting as interactions and relationships, and structural in its dealing with underlying institutional mores and systems-based practice. We evaluate (consciously and subconsciously) our students and ourselves within these frameworks.

It is this hidden curriculum that is most influenced by the humanities, the art of medicine, and our approach to one another as well as to our patients.

It is of note that though the ACGME does have one of the six core competencies for resident physicians completely focused upon “professionalism, as manifested through a commitment to carrying out professional responsibilities, adherence to ethical principles, and sensitivity to a diverse patient population” the other 5 competencies embody nuances of the humanities as well. Both Osler and Flexner would be proud.

Regulatory Bodies aim to assure wellness and wholeness at each stage of professional development: LCME (medical students), ACGME (resident/fellow wellness), AMA (the entire spectrum from medical student to retired physician). Various medical schools and residency training programs have their own wellness programs and committees.

Despite this, there is data showing increased burnout, cynicism, and suicide in physicians and physician learners as well as increased attrition from practice. Additionally, there is data showing intense dissatisfaction with delivery of medical care:

• Electronics (EHR with cut and paste data that that replicates error)
• Decreased performance of physical exams as the patient is treated as an “icon” - with the computer the focus of attention and little to no eye contact with the patient.
• Increased iatrogenic error and events (despite current focus on Q/I),
• Increasing costs, and even fraud as a result of template driven check boxes
• Business model of physicians as RVU-accountable employees subservient to the directives of non-physician bosses. (“The practice of medicine is an art, not a trade; a calling, not a business; a calling in which your heart will be exercised equally with your head.” William Osler)

How then to heal the healers? How to teach those intangible aspects of our humanity that are learned from birth? There is a learning theory used in elementary childhood education known as the Chickering Model of student development. It addresses seven pertinent areas: competence, emotions, autonomy, identity, interpersonal relationships, purpose, and integrity. Perhaps these qualities might be brought to conscious awareness and cultivated, with students’ becoming mirrors for one another and encouraging mutual trust and care.

Another theory, the Discovery Model of Learning was developed in 1960 by an educational psychologist, Jerome Bruner who later was invited to collaborate with the National Academy of Science (NAS). It is grounded in the use of inquiry, past experiences and knowledge, and intuition, creativity, and imagination. This theory seems to be undergird the aforementioned Remen “Healer’s Art Program” though not by intent. Dr Remen used no conscious educational theory but developed the program “intuitively” and “organically.”

This constructivist model of education was of interest to the NAS as a method to enhance creative scientific inquiry. It is a model of early childhood development being used for scientists in the cultivation of analytics and creativity. Perhaps these learning methods for professional development might foster benefits for adult learners with the following results:

A positive learning environment and improved physician well being

Improved diagnostic skills as result of improved analytical thinking

Improved patient care and healing

These results are *especially* needed in the context of an increasingly mechanistic and RVU driven climate, and can provide a spirit of hope to improve the well being of ourselves and those we serve.

## “Cure sometimes, treat often, comfort always.” Hippocrates

These approaches hold great potential to bring meaning to our daily lives as we seek to educate our young physicians and to heal our patients, increasing our diagnostic skills leading to accurate diagnoses and healing connections. As our scientific knowledge (and access to it) continues to expand, we will be enabled to translate into practice -- as well as to interpret -- knowledge, skills, and attitudes that have been cultivated in a spirit of humility and grace through and over time.

We will remember our professional lineage.

We will remember who we are, whence we have come, and what we are called to do.

It is our wounds that bind us together and bring us into the light.

## Take Home Messages

• “Past is prologue”: The challenges of balancing the art and science of medicine have prevailed over generations and will continue to be a challenge especially in the context of bioinformatics and mechanization.
• The art and science of medicine are not dichotomous but synergistic.
• Physician Burnout is a serious problem that can be helped through cultivation of core values and connection with fellow man.
• Our professional lineage, beginning with Hippocrates, the father of modern medicine, is worthy of rememberance as this is the legacy of our students.

## Notes On Contributors

Sarah Rice Sandlin Stender, MD, FAAP, FSAHM is an adolescent and young adult physcian who is just completing the Teaching Scholars Program at UCSF San Francisco. This paper is the culmination of many years of reading and thought on the subject of the importance of cultivation of core values and the spritual component of healing. She has been in past years a Co-Leader of the Spirituality Special Interest Group of the Society for Adolescent Health and Medicine. She was an English/Mathematics major at Vanderbilt Unversity. Currently, she is a Professor of Clinical Pediatrics and Director of the Adolescent and Young Adult division of the Department of Pediatrics, UCSF in Fresno, CA.

Sarah Rice Stender is a fourth year medical student at LSU Health Science Center in New Orleans. She too is a graduate of Vanderbilt Unversity with a BA, having majored in Political Science, Religious Studies with minor in English Literature, two favorite writers being Shakespeare and Dostoevsky.
