# Supplementary material for: The Healer's Art: Remembering Our Professional Lineage in Community through the Cultivation of Individual Core Values
Source: MedEdPublish (2016). 2018 Sep 4;7:194. [Version 1] doi: 10.15694/mep.2018.0000194.1 (PMC10701806; doi:10.15694/mep.2018.0000194.1)
Supplement: Peer review history

# Reviewer report: Reviewer response for version 1

**DOI:** 10.21956/mep.19668.r29366

**Published:** 2018-09-11

**Authors:** David Taylor

**Affiliations:** 1 Gulf Medical University, Ajman, UAE

This review has been migrated. The reviewer awarded 5 stars out of 5 I came to this paper at the end of a difficult day, and I am so glad that I read it. Amid the pressures of our day to day world it is often difficult to retain the vision of why we are here. The increasing reliance on technology has, according to the authors, distanced the physician from the patient, and yet, it has given more tools and information at our fingertips that our forebears could ever have imagined. Observing the concerns through the lens of three landmark papers was an inspired device. Of course, other articles could have been chosen, but the authors have woven them into their narrative well. I would recommend this as a “come back to” article whenever one is faced with appraisal!

Reviewer Expertise:

NA

No decision status is available

# Reviewer report: Reviewer response for version 1

**DOI:** 10.21956/mep.19668.r29365

**Published:** 2018-09-07

**Authors:** P Ravi Shankar

**Affiliations:** 1 American International Medical University

This review has been migrated. The reviewer awarded 5 stars out of 5 Technology will play an increasingly important role in twenty-first century medicine. The development of artificial intelligence, big data and algorithms will play an increasingly important role in diagnosis and treatment. As the authors mention doctors increasingly work under non-medical personnel whose primary motive is profit maximization and resource optimization. These are a few of the challenges facing medicine today. Another factor I often think about is the ever increasing amount of knowledge and skills which a medical student has to master. Is four years enough to study all this? Medicine is already one of the longest courses and can we add more years to medical training. I agree with the authors that doctors interact more with their laptops, computers and diagnostic equipment than with the patient. Performing a physical examination is becoming rarer. Also robots and machine intelligence will play an increasingly important role in the future. They do not tire, can have unlimited working hours and are getting ever better at their jobs. What does this dominance of technology mean for the doctor of tomorrow? This interesting paper raises some disturbing questions while emphasizing the importance of the humanities and the arts in modern medical education.

Reviewer Expertise:

NA

No decision status is available

# Reviewer report: Reviewer response for version 1

**DOI:** 10.21956/mep.19668.r29367

**Published:** 2018-09-04

**Authors:** Trevor Gibbs

**Affiliations:** 1 AMEE

This review has been migrated. The reviewer awarded 5 stars out of 5 A very interesting paper with some excellent references to very relevant papers. One cannot avoid the passion that comes through this paper and how the authors have brought it up to date in recognising the present day situations of physician burn-out, stress and suicide in younger physicians.A must read I believe for those involved at senior levels in Universities and Medical Schools as well as those occupying positions of organisational power within our many hospitals.I would like to repeat one of the Take Home Messages- The art and science of medicine are not dichotomous but synergistic, and add my own extra- understanding doctors make for good patients.

Reviewer Expertise:

NA

No decision status is available
